# 

**Published:** 1905-03-15

**Authors:** 


### CONTENTS.

Event and Comment - - - - - - - - - - - 109
Sanitary Artificial Dentures, - - - By N. S. Hoff, D.D.S. 118
Abstract of a Report on Dental Anesthesia, - By Dr. E. Sauvez 124
Errors of Diet, - - - - - - By Robert A. Gunn, M.D. 134
The Treatment of Alveolar Abscess, By J. P. Buckley, Ph. G., D.D.S. 139
Water-Drinking Indications and Contra-indications - - - - 142
Announcements - - - - - - - - - - - - - 145
Obituary - - - - - - - - - - - - - 149
Indents - - - - - - - - - - - - - 150

